# Social Media Mentions of Electronic Nicotine Delivery Systems (ENDS) Battery-Related Overheating, Fires, and Explosions: Findings from a Pilot Study

**DOI:** 10.3390/ijerph16081308

**Published:** 2019-04-12

**Authors:** Sarah Trigger, Blair Coleman

**Affiliations:** Office of Science, Center for Tobacco Products, U.S. Food and Drug Administration, Silver Spring, MD 20993, USA; blair.coleman@fda.hhs.gov

**Keywords:** social media, electronic nicotine delivery systems (ENDS), overheating, fires, explosions

## Abstract

Serious injuries may result from electronic nicotine delivery systems (ENDS) battery malfunctions, including overheating, fires, and explosions (O/F/E). This pilot study assessed the usefulness of social media monitoring as a tool for gathering information surrounding ENDS battery O/F/E, including changes in the volume and nature of social media mentions over time. Brandwatch, a social media monitoring tool, was queried to examine ENDS battery-related O/F/E over a one-month period, annually, from 2013–2017. Two researchers qualitatively coded the social media mentions for relevance and coded the relevant mentions by event type and theme. The total number of mentions coded as relevant (*n* = 947) for the one-month period increased each year. Mentions of first-person events were relatively infrequent (3.6% of relevant mentions), while mentions describing events that happened to someone else increased over time (60.4% of relevant mentions). A relatively small proportion of mentions expressed concern around a potential event and advice on how to prevent future events (4.8% and 10.5% of relevant mentions, respectively). Findings suggest that social media mentions around ENDS battery O/F/E events have increased over recent years. Social media monitoring can complement traditional surveillance systems to elucidate the extent to which ENDS O/F/E events are occurring.

## 1. Introduction

As of 2015, nearly eight million US adults were current users (every day or some days) of electronic nicotine delivery systems (ENDS) [1]. ENDS batteries may be defective or may malfunction, resulting in overheating, fires, and explosions (O/F/E), which in turn can cause serious bodily injury and property damage [2]. Data from local and regional burn registries in the US have documented cases of ENDS-related burn injuries dating back to 2009 [3,4], and a review of events reported to federal agencies, the scientific literature, and media outlets identified 34 unique burn injuries to ENDS users and five unique injuries to non-users from 2009–2015 [5]. Only two published studies have documented nationally-representative estimates of ENDS battery-related burn injuries at the population level, estimating approximately 2000 injuries presented in US emergency departments from 2015–2017 [6,7].

While the most serious cases of ENDS battery-related injuries in the US have been reported via case reports and media outlets, less severe ENDS O/F/E may go unreported. To address this gap, a pilot study was conducted to assess the utility of social media monitoring for gathering information surrounding ENDS battery O/F/E.

## 2. Materials and Methods

### 2.1. Data Source

Brandwatch is a social media monitoring company that licenses software to collect publicly available data from online platforms, including Twitter, Facebook, Instagram, select blogs (e.g., Tumblr), forums (e.g., Reddit), news sites (e.g., MSN), and video sites (e.g., YouTube).

We queried Brandwatch’s social media listening tool to capture social media mentions of ENDS O/F/E from 1–31 July each year from 2013–2017 (July was the most recent month available at the time this study began). A single month (July) across years was selected to provide a snapshot of the online discussion of ENDS O/F/E and to keep the number of mentions manageable for manual qualitative coding. The query, developed in conjunction with a contractor (Apprio, Inc., Washington, DC, USA), included product terms (e.g., ecig, vape pen, vaping, e-liquid, battery, cartridge, mod) in conjunction with event terms (e.g., blow up, overheat, on fire, singe, failure, explode, burn) to capture ENDS O/F/E events (Appendix A). In an attempt to isolate social media mentions in the US, all mentions were filtered for those posted in English and those posted from the US (e.g., using time zone information and IP addresses); however, it is possible the filter may have inadvertently captured posts not made in the US or posts with content not about US people and events.

### 2.2. Data Measurement

The two categories of interest were: (1) The type of event described in the social media mention and (2) the theme of the social media mention.

For event type, mentions were examined for the presence (yes) or absence (no) of description of first-person events, coded as “event/self,” and description of events that occurred to someone else, coded as “event/other”.

For theme, mentions were examined for the presence (yes) or absence (no) of expression of concern about potential ENDS battery-related events, coded as “theme/concern,” and expression of practices to prevent events from occurring, coded as “theme/prevent”.

The four codes that comprised the coding scheme were not mutually exclusive.

### 2.3. Data Coding and Analysis

The two researchers independently coded all social media mentions using a thematic approach [8] in three stages: (1) Social media mentions were examined for relevance to ENDS battery O/F/E (relevant example: “A vape pen burned my mouth”; non-relevant example: “I burn out two vape pens every day”); (2) the dataset was checked for duplicate mentions, which were removed from the dataset if they included verbatim text of a preceding mention and were posted by the same author on the same day (possibly indicating that the system had erroneously reported repetitive data); and (3) relevant mentions were coded based on event type (“event/self” and “event/other”) and theme (“theme/concern” and “theme/prevent”).

Additionally, Brandwatch provided categorizations for page type (e.g., Twitter, forum).

Data were analyzed by year, examining the proportion of each event type, mention theme, and page type among the relevant posts in July of a given year.

Coders met periodically to discuss and resolve discrepancies. Inter-rater reliability was 98.9% for determining whether mentions were relevant [9].

## 3. Results

Of the 13,126 mentions reviewed for this study, 1117 (8.5%) were coded as relevant. The mentions coded as irrelevant include non-relevant topics and ads. Of the 1117 relevant mentions, 947 (84.8%) were found to be non-duplicated. However, multiple non-duplicated mentions may still refer to a single event. All results that follow refer to the 947 non-duplicated relevant mentions.

### 3.1. Changes in Volume of Mentions over Time

Data collected from the month of July in the years 2013–2017 showed an increase in the total number of social media mentions related to ENDS O/F/E (Table 1). Specifically, the total number of relevant mentions increased from 65, in 2013, to 357, in 2017. Across each year, a gradual increase in total relevant mentions was observed, except for 2013–2014.

### 3.2. Changes in Mention Type and Theme over Time

Social media mentions of first-person events (“event/self”) were relatively infrequent, ranging from two mentions in 2013 to 11 mentions in 2017, comprising 3.6% of the relevant mentions. Examples of “event/self” mentions included: “My vape pen just caught on fire” and “My ecig exploded in my pocket and now my entire thigh is covered in strong nicotine juice”.

Mentions describing events that happened to someone else (“event/other”) comprised 60.4% of all mentions. “Event/other” mentions generally increased over time from 57, in July 2013, to 225, in July 2017 (except for a decrease from 2013–2014). Examples of “event/other” mentions included: “Corona couple sues after e-cigarette battery explodes in car” and “Was sitting in my night class and a guy’s ecig started his pants on fire lol…”

Relevant mentions that expressed concern around a potential O/F/E event (“theme/concern”) increased from 1, in 2013, to 23, in 2017, though these were generally uncommon, comprising only 4.8% of relevant mentions. Examples of “theme/concern” mentions included: “Charging my ecig makes me paranoid. It’s totally gonna blow up. Irrational, but there’s still a pillow between it and me.” and “Thes[e] ecig fire explosions make me very concerned! What does the #vape industry do to guarantee maximum security?”

Relevant mentions expressing prevention strategies (“theme/prevent”) increased from zero, in 2013, to 52, in 2017 (except for a decrease from 2015–2016), and comprised 10.5% of mentions. Examples of “theme/prevent” mentions included: “Be careful of leaving your mod/batteries inside the car on a hot summer day! #vape #vapesafe” and “All you have to do is keep your e-cig apart from your keys or coins due to the lithium battery. I’ve been vaping for over 7 years and never had a battery explode”.

As noted earlier, the four main coding categories were not mutually exclusive. For example, a single mention could be both about an event that occurred to someone else (“event/other”) as well as expressing concern about an event (“theme/concern”), as with the mention: “A West Fargo man says his vape pen burst into flames and burned his finger, and he wants others to know the dangers of the devices.” Notably, one in four mentions (24.6%; *n* = 233) did not meet the criteria for any of the four categories. These relevant mentions included sarcastic comments (e.g., “Do cars overheat anymore or is it just always a vape pen”) and non-specific mentions around ENDS O/F/E (“After just 35 explosions, Samsung globally recalled Note 7… Why no recall of exploding #ecigs?”).

## 4. Discussion

Findings from this pilot study suggest that conversation volume around ENDS battery O/F/E events has increased over recent years. Moreover, the nature of social media mentions of ENDS O/F/E has evolved over time, such that an increase was observed in mentions of events occurring to others. There were modest increases in total mentions expressing concern for future events or ways to prevent events; however, the total number of mentions for the concern and prevention themes remained relatively infrequent over time (4.8% and 10.5% of total relevant mentions, respectively). Total mentions relating to events that occurred to oneself remained comparatively low over time (3.6% of total relevant mentions).

To date, much of the published literature on this topic has reported local or regional cases documenting severe burn injuries [3,4] or national publicly reported incidents [2], with only two reporting nationally representative estimates of ENDS battery-related burn events [6]. Findings from this study complement previous studies [2,3,4,5,6,7] by providing information on consumer reports of ENDS battery-related O/F/E, demonstrating that social media can provide insight into less serious events and a better sense of the spectrum of outcomes. The current study findings also extend prior research on this topic by providing preliminary evidence suggesting that ENDS consumers may be expressing concern about potential events online, as well as discussing ways in which they may be able to avoid future events. Such information lends support for the development of future education campaigns on ENDS battery safety and may also inform ongoing efforts to protect the public from battery-related O/F/E events [10]. Lastly, enhanced surveillance that draws from social media may expand our understanding of ENDS battery-related O/F/E events at the population level, including events that are both severe and acute in nature.

### Limitations

Brandwatch, as with other social media listening tools, is unable to capture all social media mentions due to privacy settings across platforms; thus, these data are not representative of all online conversations around ENDS battery O/F/E and cannot be used to infer prevalence of O/F/E events. Additionally, while we attempted to target social media mentions posted in the US via a Brandwatch-created filter, it is possible that the query inadvertently captured mentions from other countries. Brandwatch data collected prior to the query creation may be incomplete due to the tool collecting historical data vs. collecting data in real-time (whereas this pilot project query was created in 2017, a parent query began collecting social media data in September 2015). As a pilot study, investigators selected the most recent month (July) with available data to use for inclusion across study years; conclusions may change with expanded search criteria, restrictions to unique stories, or time of year.

## 5. Conclusions

Findings suggest that social media discussions surrounding ENDS battery-related O/F/E are increasing over time, particularly for mentions of events occurring to others. Results from this pilot study support the use of social media data as a useful source of information to complement other surveillance and monitoring efforts related to ENDS battery-related O/F/E events, providing insight into less serious events that may not be reported by other channels.

## Figures and Tables

**Table 1:** Summary of relevant social media mentions collected in the electronic nicotine delivery systems (ENDS) overheating, fires, and explosions (O/F/E) query, by event type, mention theme, and page type, 2013–2017.

Categories of Interest	2013 (1–31 July) (*n* = 65)	2014 (1–31 July) (*n* = 44)	2015 (1–31 July) (*n* = 174)	2016 (1–31 July) (*n* = 307)	2017 (1–31 July) (*n* = 357)	Total (*n* = 947)
	*n* (%)	*n* (%)	*n* (%)	*n* (%)	*n* (%)	*n* (%)
**Event Type ^1^**						
Event/Self	2 (3.1)	7 (15.9)	7 (4.0)	7 (2.3)	11 (3.1)	34 (3.6)
Event/Other	57 (87.7)	13 (29.5)	76 (43.7)	201 (65.5)	225 (63.0)	572 (60.4)
**Mention Theme ^1^**						
Theme/Concern	1 (1.5)	1 (2.3)	10 (5.7)	10 (3.3)	23 (6.4)	45 (4.8)
Theme/Prevent	0 (0.0)	4 (9.1)	27 (15.5)	16 (5.2)	52 (14.6)	99 (10.5)
**Page Type ^2^**						
Blog	0 (0.0)	0 (0.0)	8 (4.6)	4 (1.3)	3 (0.8)	15 (1.6)
Facebook	0 (0.0)	0 (0.0)	0 (0.0)	8 (2.6)	6 (1.7)	14 (1.5)
Forum	0 (0.0)	0 (0.0)	28 (16.1)	16 (5.2)	68 (19.0)	112 (11.8)
General ^3^	0 (0.0)	0 (0.0)	2 (1.1)	15 (4.9)	3 (0.8)	20 (2.1)
Instagram	0 (0.0)	0 (0.0)	0 (0.0)	1 (0.3)	3 (0.8)	4 (0.4)
News	0 (0.0)	0 (0.0)	10 (5.7)	30 (9.8)	14 (3.9)	54 (5.7)
Twitter ^4^	65 (100.0)	44 (100.0)	126 (72.4)	233 (75.9)	253 (70.9)	721 (76.1)
Video	0 (0.0)	0 (0.0)	0 (0.0)	0 (0.0)	7 (2.0)	7 (0.7)

^1^ Codes are not mutually exclusive. ^2^ Mentions were categorized by the social media platform source of posting. This categorization was provided by Brandwatch. Categories are mutually exclusive. ^3^ The “general” category on Brandwatch encompasses sites not captured by another category, e.g., Google+. ^4^ Relevant mentions were only captured from Twitter in July 2013 and 2014.

## Appendix A. ENDS O/F/E Query

((ecig* OR “e pipe” OR epipe* OR “vape pen” OR “vape pens” OR (personal NEAR/0f vap*) OR vaping OR “e-liquid” OR eliquid* OR ejuice* OR electroniccig* OR atomizer OR battery OR cartomizer OR cartridge OR clearomizer OR coils OR tank OR mod OR mods OR “18650” OR “26650” OR “USB charger” OR vaporizer) NEAR/7 (adverse NEAR/0 event) OR blister* OR “blow up” OR “blew up” OR burst OR “on fire” OR flame OR flames OR overheat* OR scald* OR scorch* OR sear* OR singe* OR smolder* OR pregn* OR harm* OR defect* OR recall* OR failur* OR quality OR malfunct* OR complaint* OR contaminat* OR explod* OR explos* OR burn* OR injur* OR poison* OR overdos* OR (accident* NEAR/1 ingest*) OR (child* NEAR/1 expos*) OR sick OR (health NEAR/3 (problem* OR issue*) OR toxin OR toxins) OR (Apollo OR Epuffer OR Eversmoke OR “Halo Cigs” OR Juul OR “Mig Vapor” OR Smoketip OR “South Beach Smoke” OR “V2 Cigs” OR Vapor4Life OR Vaporfi OR “White Cloud”)) NEAR/15 ((adverse NEAR/0 event) OR blister* OR “blow up” OR “blew up” OR burst OR “on fire” OR flame OR flames OR overheat* OR scald* OR scorch* OR sear* OR singe OR smolder* OR pregn* OR harm* OR defect* OR recall* OR failur* OR quality OR malfunct* OR complaint* OR contaminat* OR explod* OR explos* OR burn* OR injur* OR poison* OR overdos* OR (accident* NEAR/1 ingest*) OR (child* NEAR/1 expos*) OR sick OR (health)) NEAR/3 ((problem* OR issue*) OR toxin OR toxins). The ENDS O/F/E query excluded: “apollo 13” OR (burst NEAR/2f flavor) OR EpiPen OR EpiPens).
